# Water-Resistant Thermoelectric Ionogel Enables Underwater Heat Harvesting

**DOI:** 10.3390/polym15071746

**Published:** 2023-03-31

**Authors:** Long Li, Huijing Li, Junjie Wei, Rui Li, Jiale Sun, Chuanzhuang Zhao, Tao Chen

**Affiliations:** 1 School of Material Science and Chemical Engineering, Ningbo University, Ningbo 315211, China; 2 Key Laboratory of Marine Materials and Related Technologies, Zhejiang Key Laboratory of Marine Materials and Protective Technologies, Ningbo Institute of Material Technology and Engineering, Chinese Academy of Sciences, Ningbo 315201, China; 3 University of Chinese Academy of Sciences, Beijing 100049, China

**Keywords:** gels, water resistance, ionic thermoelectrics, heat energy, underwater environment

## Abstract

The energy crisis is one of the most critical and urgent problems in modern society; thus, harvesting energy from ubiquitous low-grade heat energy with thermoelectric (TE) materials has become an available strategy in sustainable development. Recently, emerging ionic TE materials have been widely used to harvest low-grade heat energy, owing to their excellent performance in high ionic Seebeck coefficient, low thermal conductivity, and mechanical flexibility. However, the instability of ionic conductive materials in the underwater environment seriously suppresses underwater energy-harvesting, resulting in a waste of underwater low-grade heat energy. Herein, we developed a water-resistant TE ionogel (TEIG) with excellent long-term underwater stability utilizing a hydrophobic structure. Due to the hydrophobic polymer network and hydrophobic ionic liquid (IL), the TEIG exhibits high hydrophobicity and antiswelling capacity, which meets the requirement of environment stability for underwater thermoelectric application. Furthermore, the water resistance endows the TEIG with great thermoelectric performances in the underwater environment, including satisfactory ionic Seebeck coefficient, outstanding durability, and superior salt tolerance. Therefore, this investigation provides a promising strategy to design water-resistant TE materials, enabling a remarkable potential in harvesting low-grade heat energy under water.

## 1. Introduction

The energy crisis is one of the most critical and urgent issues in the 21st century. As we all know, energy is the foundation for the survival and development of human society, and all human activities are inseparable from the supply of energy. Thermal energy is abundant and ubiquitous on Earth; nevertheless, only one third of it is being used through multilevel conversion [1]. Most of the remaining heat is dissipated into the external unexploited environment directly, which causes not only a serious waste of energy but environmental thermal pollution [2,3]. Moreover, the thermal energy dissipated into the external environment is mainly low-grade heat energy, which is difficult to harvest due to its low energy density and low heat utilization efficiency [4], such as industrial waste heat [5], solar light heat, and human joule heat [6]. Therefore, efficiently harvesting low-grade waste heat is of great significance for improving energy efficiency and realizing sustainable development [7,8].

Using TE material is the most effective way to harvest low-grade heat energy, because it can convert heat into electrical energy directly without any moving parts [9,10,11,12,13]. In addition, TE material is safe, reliable, emission-free, pollution-free, and has important application prospects in the fields of the self-powered Internet of Things, 5G communication, electronic skin, deep space exploration, health monitoring, etc. [14,15,16,17]. Compared with the traditional electronic TE materials, the ionic TE materials based on ion charge carriers, including liquid electrolytes, ionic hydrogels, and ionogels [18,19,20,21,22], have attracted increasing attention and made great progress recently because of their high ionic Seebeck coefficients, low thermal conductivity, and excellent mechanical flexibility. For example, Ouyang et al. demonstrated an environment-benign flexible quasi-solid TEIG with giant ionic Seebeck coefficient (~26.1 mV/K) and ultrahigh thermoelectric properties, including a high ionic conductivity of 6.7 mS/cm, a low thermal conductivity of 0.176 W/(m·K), and a thermoelectric figure of merit of 0.75, exhibiting great thermoelectric properties for the conversion of intermittent heat into electricity [23]. However, the existing TE materials can only work in a low-humidity air environment, because they are mostly aqueous solutions or have water solubility with ions diffusion leakage and swelling behavior in underwater environments. Although packaging is an alternative strategy to achieve underwater application, the packaging layer will inevitably disturb the flexibility and heat transfer [24,25,26]. Developing inherently water-resistant ionic TE material is still a significant challenge for underwater heat harvesting.

**Figure 1:**
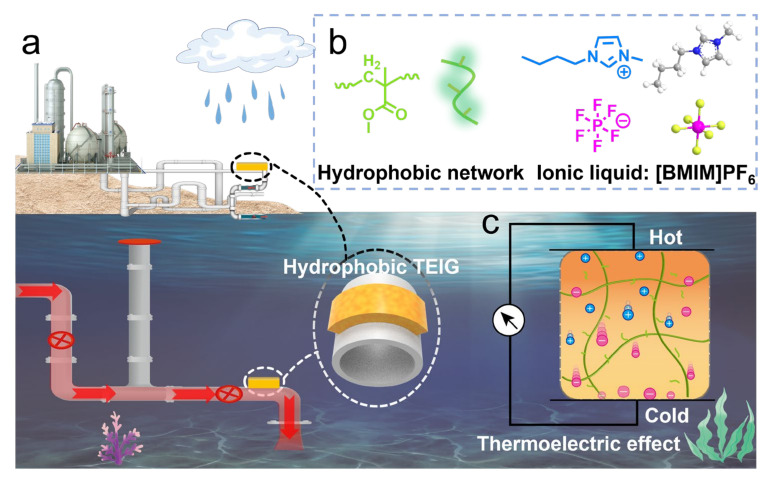
Schematic of the fabrication and application of the hydrophobic ionic thermoelectric ionogel: (**a**) Application of TEIG in submarine environment and rainy environment. (**b**) Hydrophobic monomer and hydrophobic IL. (**c**) Structure schematic of hydrophobic ITEC.

Interestingly, hydrophobic materials can eliminate the interference of water molecules effectively, owing to their low surface energy and moisture absorption, providing a possibility for realizing underwater stable application [27,28]. Inspired by this, in this work, we designed a delicate TEIG that is suitable for aquatic environments through one-step polymerization of hydrophobic monomer (Methyl methacrylate, MMA) in hydrophobic IL solvent (1-Butyl-3-methylimidazolium hexafluorophosphate, [BMIM]PF_6_). As we expected, the hydrophobic structure endows ionogel with excellent water resistance and stable underwater thermoelectric performance, remaining stable in both dry and underwater environments without ion leakage and gel swelling. Therefore, the water-resistant TEIG exhibits a profound potential in the field of harvesting low-grade heat energy in a submarine environment and rainy environment (Figure 1).

## 2. Materials and Methods

### 2.1. Materials

Methyl methacrylate (MMA, 99.5%), 1-Butyl-3-methylimidazolium hexafluorophosphate (BMIMPF_6_, 97%), Ethylene glycol dimethacrylate (EGDMA, 98%), and 2, 2-diethoxyacetophenone (DEAP, ≥95%) were bought from Sigma-Aldrich (Shanghai, China) (all chemicals used are shown in Figure S1).

### 2.2. Preparation of TEIG

The TEIG was prepared by the following method: firstly, different volume ratios of IL and MMA monomer (4:1, 4:2, 4:3, 4:4) were dissolved, with the volume of BMIMPF_6_ fixed at 12 mL at 25 °C to make a homogeneous aqueous solution. A total of 0.1 wt % EGDMA crosslinker and 0.5 wt % DEAP photoinitiator were added. Note that there is no water used in the preparation process; the above mass percentage is calculated by the mass of monomer MMA, not by the mass of water. The solution was dealt with ultrasound for 10 min before being poured into a mold composed of two quartz glass and a silicone frame with an inner size of 50 mm × 50 mm × 4 mm. Then, the TEIG was formed by photoinitiation polymerization by ultraviolet lamp (50 W) for 3 h.

### 2.3. Preparation of ITEC

The TEIG element (size is 10 mm × 10 mm × 4 mm) was sandwiched by copper foil to form the ITEC, on which copper foil extended as wireway to connect an electrochemical workstation. An ITEC device with enhanced thermal voltage was obtained by connected in series.

### 2.4. Tensile Mesurement

The tensile stress–strain properties of the TEIG were measured by an electronic universal testing machine (Z1.0, Zwick, Ulm, Germany). The strip samples, 5 mm in width and 1 mm in thickness, were tested at room temperature with 50 mm/min tensile speed. The strain was calculated as the following equation:

(1) $$(L-L_{0})/L_{0}\times 100\%$$

where L was the length and $L_{0}$ was the initial length.

### 2.5. Water Contact Angle (WCA) Measurement

The water contact angles were measured by the OCA25 Contact Angle Measuring System (Dataphysics, Filderstadt, Germany). A 2 µL water droplet was carefully deposited on TEIG surfaces using a syringe.

### 2.6. Swelling Behavior Measurement

The swelling behavior was studied by soaking the TEIG in deionized water and weighting the ionogel samples every 12 h. The swelling ratio (*SR*) was calculated by the following equation:

(2) $$SR=\frac{M_{S}-M_{0}}{M_{0}}$$

where $M_{S}$ is the swollen weight and $M_{0}$ is the initial weight of the TEIG.

### 2.7. Thermal Stabilities Measurement

The thermal stabilities were measured by a TGA (TGA 8000-Spectrum two-Clarus SQ8T, PerkinElmer, Waltham, MA, USA), in which the temperature increasing speed was 5 °C min^−1^.

### 2.8. Ionic Conductivity Measurement

The room-temperature ionic conductivity (*σ*) of the TEIG was measured by AC impedance technique. In this experiment, the samples were sandwiched between two cooper electrodes without an air gap between samples and electrodes.

The measurements were carried out by an electrochemical workstation (CHI660E, CH Instruments Co., Ltd., Austin, TX, USA) in the frequency range of 0.1 Hz to 100 kHz. The intersection of the curve at the real part was taken as the bulk resistance of the TEIG electrolyte (*R*), and the ionic conductivity of the sample was calculated according to the following equation:

(3) $$\sigma =\frac{d}{RS}$$

where *d* is the thickness of the TEIG electrolyte and *S* is the electrode area. The resulted real (*Z′*) and imaginary parts (*Z″*) of impedance at different frequencies were recorded as a Nyquist plot. The TEIG sample (20 mm × 20 mm × 1 mm) was sandwiched between two cooper electrodes (30 mm × 20 mm)

### 2.9. Ionic Seebeck (S_i_) Coefficient Measurement

The Si coefficient was measured under ambient conditions by homemade equipment, as shown in Figure S2. It consisted of two Peltier devices (TEC1-19906 by Beijing Geshang Electronic Pte. Ltd., Beijing, China) affixed on an alumina heat sink. The temperature difference (Δ*T*) across the sample was detected with two K-type thermocouples thermometer (Tronovo TR6601, Suzhou, China), which have a diameter of 0.13 mm, and the thermovoltage output (Δ*V*) was measured with Chenhua CHI660E electrochemical workstation. For each sample, the Δ*V* values were measured at five different Δ*T* values. The *Si* coefficient was derived through the best linear fitting of the Δ*V*-Δ*T* plots, and the *Si* was calculated in terms of the equation below:

(4) $$S_{i}=\frac{-(V_{h}-V_{c})}{\left(T_{h}-T_{c}\right)}=\frac{\Delta V}{\Delta T}$$

where *V_h_*, *V_c_*, *T_h_*, and *T_c_* are the voltages and temperatures of the hot and cold terminals, respectively, Δ*V* is the open-circuit voltage difference, and Δ*T* is the temperature difference measured by the K-type thermocouples. The thickness of TEIG was measured by a micrometer. The size of the glass was 75 mm × 25 mm, and the size of TEIG was 40 mm × 10 mm × 2 mm.

### 2.10. Thermal Conductivity Measurement

Thermal diffusivity ($D_{th}$) and specific heat capacity ($C_{P}$) were measured by a laser flash method (LFA 467, Netzsch, Waldkraiburg, Germany). Thermal conductivity was calculated by the following equation:

(5) $$\lambda =\rho D_{th}C_{P}$$

where ρ represents the density, $D_{th}$ is the thermal diffusivity, and $C_{P}$ is the specific heat capacity.

### 2.11. The Thermal Power Figure of Merit (ZT) and the Power Factor (PF)

The dimensionless thermal power Figure of merit (*ZT*) [29,30] can be calculated by the equation:

(6) $$ZT=\frac{{S_{i}}^{2}\sigma }{\lambda }T$$

where *S_i_* represents the ionic Seebeck coefficient, *σ* represents the ionic conductivity, *λ* is the thermal conductivity, and *T* is the absolute temperature. A high *ZT* value indicates a high thermoelectric conversion efficiency. The power factor (*PF*) [9,31] can be calculated by the equation:

(7) $$PF={S_{i}}^{2}\sigma$$

where the *PF* is also an index to measure the energy conversion efficiency of thermoelectric materials, and a high *PF* means a high thermoelectric conversion efficiency.

### 2.12. Density Functional Theory (DFT) Calculations

To calculate the interaction energy between BMIMPF_6_ and NaCl, we performed density functional theory (DFT) calculations and adopted the local density approximation (LDA-PWC). Materials studio software was used for all calculations, and the BUGS algorithm was used to optimize the geometric structure to make the structure reach the most stable state. The convergence standard (SCF tolerance) of the self-consistent field is 1 × 10^−5^, the BASIS set is DND, and the atomic orbital stage radius is 3.5 angstroms. It should be noted that the model is composed of two ions, and the interaction between them was studied. The interaction energy of the interaction intensity of each component in the system is calculated using the interaction energy formula:

(8) $$Eads=E_{AB}-\left(E_{A}+E_{B}\right)$$

where *E_AB_* and *E_A_*/*E_B_* are the total energy of the whole system and the energy of each component in the system, respectively. According to the above definition, negative *Eads* correspond to the interaction energy between components. The more negative *Eads*, the stronger the interaction in the system.

## 3. Results and Discussion

### 3.1. Mechanical Properties of Ionogels

Considering that polyacrylates are not only a kind of common hydrophobic polymer but also have outstanding water resistance, MMA was selected as the hydrophobic monomer to form the hydrophobic polymer network of ionogel. Additionally, BMIMPF_6_ was used as solvent and charge carrier due to its hydrophobicity (Figure S3), high ionic conductivity, and good thermoelectric properties. Utilizing a one-step photopolymerization method, the fully hydrophobic ionogel was successfully fabricated with the cross-linker (Ethylene glycol dimethacrylate, EGDMA) and photoinitiator (2, 2-Diethoxyacetophenone, DEAP) (all chemicals used are shown in Figure S1). To study the effect of hydrophobic polymer content on the hydrophobicity and thermoelectric properties of ionogel, a series of hydrophobic ionogels with various volume ratio of IL and MMA (including 4:1, 4:2, 4:3, and 4:4) was prepared, and the weight percentage of cross-linker and photoinitiator relative to the amount of MMA are fixed at 0.1% and 0.5%. After polymerization, there was no monomer residue in the obtained ionogel (Figure S4).

**Figure 2:**
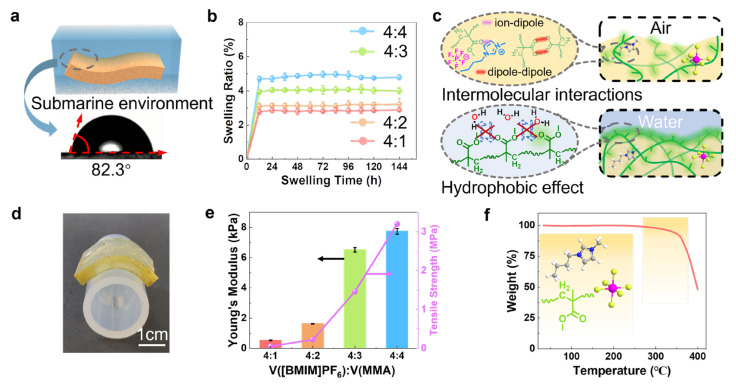
Hydrophobicity and environment stability of hydrophobic TEIG: (**a**) Water contact angle of hydrophobic TEIG. (**b**) Swelling curves of the hydrophobic TEIGs with various MMA content. (**c**) Schematic of hydrophobic TEIG’s structural change after soaking in water. (**d**) Digital photo of the hydrophobic TEIG adhered to curved circular tube. (**e**) Mechanical properties of hydrophobic TEIGs with various MMA content. (**f**) Thermogravimetric curve of the hydrophobic TEIG with 20 vol % MMA.

The hydrophobicity of these TEIGs was evaluated by contact angle test. As shown in Figure 2a and Figure S5, among them, the TEIG with a volume ratio of IL and MMA of 4:1 has the largest water contact angle of 82.3°, indicating that the hydrophobic IL plays a major role in improving the hydrophobicity of ionogel compared with hydrophobic polymers. This may be caused for two reasons, one is that IL possesses higher hydrophobicity due to the rich fluorine structure, and the other is that the IL occupies a larger proportion of the surface of ionogel as the continuous phase. The good hydrophobicity provides the basis for the long-term underwater stability of ionogel. These hydrophobic ionogels with different MMA contents were soaked into water for 6 days to study their underwater stability. Although a little swelling were observed in the beginning of the water soaking treatment, there were no remarkable weight changes for all ionogel samples in the subsequent 5 days (Figure 2b). The reason for these variation trends is that the sparse hydrophobic function groups on the ionogel’s surface start to gather under hydrophobic effect when contacting with water, resulting in lower surface free energy and higher underwater stability in the later period (Figure 2c). Meanwhile, the strong hydrophobic interactions and ion–dipole interaction between IL and polymeric network also restrain the loss of ions [32]. Consequently, the IL and MMA in a volume ratio of 4:1 showed the best antiswelling performance (swelling ratio: 2.8%) and underwater stability. The above results prove that the strategy of constructing hydrophobic structures by fully hydrophobic components is effective, with the hydrophobic network inhibiting the diffusion of H_2_O and ions effectively.

Additionally, the hydrophobic TEIG is flexible. As shown in Figure 2d, the hydrophobic TEIG could adhere to a curved circular tube well, proving the feasibility of collecting heat energy from a nonplanar heat source. Moreover, the mechanical properties of the hydrophobic TEIG were quantitatively measured. With the increase in MMA content, the tensile strength and Young’s modulus of the TEIG increased significantly (Figure 2e and Figure S6). Benefitting from its good underwater stability, the hydrophobic TEIG showed a relatively stable mechanical property, even after soaking in water (Figure S7). Furthermore, the stability of hydrophobic TEIG at high temperature was studied by thermogravimetry. As shown in Figure 2f and Figure S8, it exhibited ultrahigh decomposition temperature (>250 °C), meaning that the hydrophobic TEIG can operate at high temperature, which is conducive to form high temperature differences and generate ultrahigh thermovoltage.

### 3.2. Thermoelectric Properties in Air

**Figure 3:**
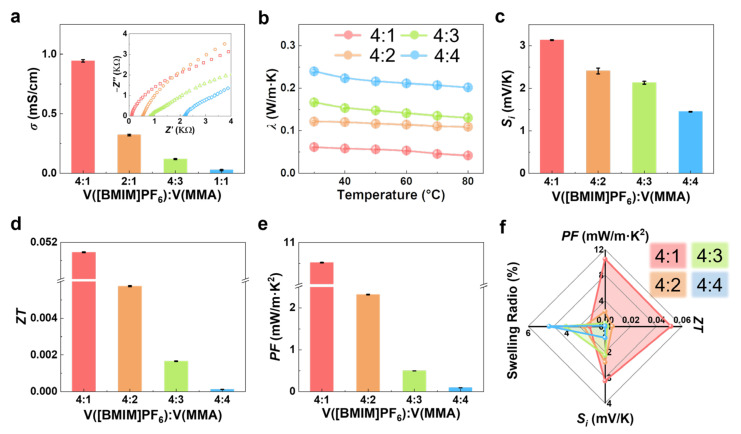
Thermoelectric parameters optimization of hydrophobic TEIG: Effect of volume ratio of IL to MMA on ionic conductivity (*σ*) (**a**), thermal conductivity (*λ*) (**b**), ionic Seebeck coefficient (*S_i_*) (**c**), power factor (*PF*) (**d**), and thermal power Figure of merit (*ZT*) (**e**) of the hydrophobic TEIGs. (**f**) Comparison of comprehensive properties of the hydrophobic TEIGs with various volume ratios of IL and MMA.

According to the definition of the thermal power Figure of merit (*ZT*), ionic conductivity (*σ*), thermal conductivity (*λ*), and ionic Seebeck coefficient (*S_i_*) are the key parameters that determine the energy conversion efficiency of thermoelectric materials. To optimize the *ZT* value of the TEIG, the thermoelectric parameters of four TEIGs with various MMA volume percentages were investigated and compared. Their ionic conductivity was measured by electrochemical impedance spectroscopy. As shown in Figure 3a, the ionic conductivity of the TEIG decreases with increasing MMA monomer content. It is generally accepted that ionic conduction is caused by the directional movement of free ions under an electric field. However, with more MMA monomer, the polymer network become denser, which reduces the space for free ion movement and reduces the conductivity. Therefore, the TEIG with an MMA volume ratio of 4:1 achieved the greatest ionic conductivity of 0.94 mS/cm among all samples. The thermal conductivities were measured by the laser flash method.

As shown in Figure 3b, when the MMA volume ratio changed from 4:1 to 4:4, the thermal conductivity of TEIGs increased from 0.061 W/(m·K) to 0.24 W/(m·K). It is commonly known that the IL ([BMIM]PF_6_) has a lower thermal conductivity than PMMA polymer, so the thermal conductivity of TEIG has a positive relation with MMA monomer content [33,34]. Additionally, the high temperature will accelerate the thermal diffusivity of ions, leading to an enhanced thermal conductivity.

As shown in Figure S2, the *S_i_* of TEIGs with different MMA content were tested. The *S_i_* decreases significantly from 3.13 mV/K to 1.5 mV/K, with the volume content of MMA rising (Figure 3c and Figure S9). The Seebeck voltage mainly relies on the difference of thermal migration rate between anions and cations, which leads to an asymmetric ion concentration distribution on two electrodes. The higher MMA content leads to a denser polymer network, i.e., fewer free moving anions and cations, resulting in lower ion concentration difference between two electrode terminals and lower *S_i_*. Additionally, according to the theory of heat transfer (Q*) introduced by Eastman, the decline in ion content will bring a decrease in Q* value, which also results in a low ionic Seebeck coefficient, since the ionic Seebeck coefficient is proportional to Q* value. Although this thermoelectric coefficient is not outstanding, it is still competitive with other ionogelsreported previously (Figure S10) [35,36,37,38,39,40].

A high *ZT* value indicates a high thermoelectric conversion efficiency. As shown in Figure 3d, when V(IL): V(MMA) reached 4:1, the TEIG had the highest *ZT*. Similarly, the power factor (*PF*) expressed by *S_i_*^2^*σ*, another key parameter to evaluate the performance of thermoelectric materials, presented the greatest value of 10.5 mW/m·K^2^ when the volume ratio was 4:1 (Figure 3e). After comprehensive evaluation of thermoelectric properties (including *S_i_*, *ZT* and *PF*) and underwater stability (swelling ratio) of the TEIGs, the ionogel with a volume ratio of 4:1 showed the best thermoelectric performance and water resistance, and it was selected as the best TE material for the later experiment (Figure 3f).

### 3.3. Thermoelectric Properties under Water

**Figure 4:**
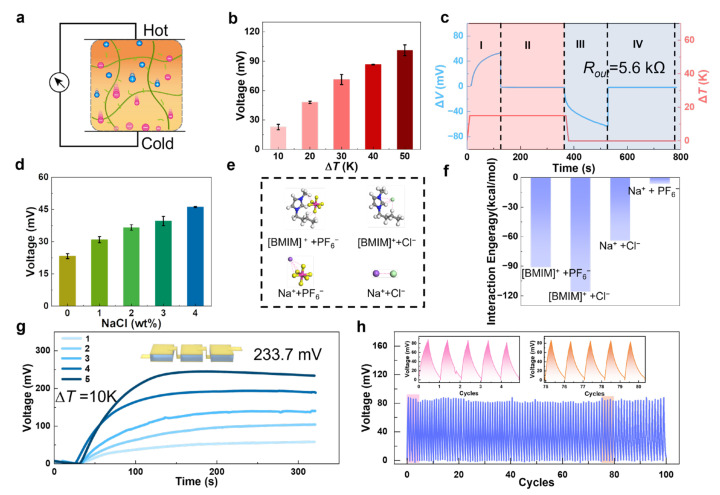
Underwater thermoelectric performances of hydrophobic ITEC: (**a**) Schematic of ion distribution of ITEC under temperature difference. (**b**) The thermovoltage of TEIG under different temperature differences in the underwater environment. (**c**) Thermovoltage profiles with an external load connected or disconnected under given temperature gradients. (**d**) Thermovoltage of ITEC in the water with different salt content. (**e**) DFT-optimized structures of different ions’ pairing, including BMIM^+^: PF_6_^−^, BMIM^+^: Cl^−^, Na^+^: PF_6_^−^, and Na^+^: Cl^−^. (**f**) DFT calculations evaluating binding energy between different ions’ pairing. (**g**) Thermovoltage value of TE device formed by different number of TE elements in series. (**h**) Cyclic power generation test of the TE device composed of three TE elements under intermittent temperature difference (10 K) in the underwater environment.

Reasonable thermoelectric performance and excellent water resistance enable TEIG to harvest heat into electric energy as ionic thermoelectric capacitors under water. As shown in Figure 4a, an underwater ionic thermoelectric capacitor (ITEC) was fabricated by the optimized TEIG. When there is no temperature difference between two terminals of the TEIG, the anions and cations are uniformity dispersed in it, causing no voltage to be generated. Contrarily, when there is a temperature gradient in the TEIG, the anions and cations move from the hot terminal to the cold terminal due to the ion thermal diffusion (Soret) effect. Because the volume and quality of the anions and cations are different, their migration rates are distinct, leading to the uneven distribution of anions and cations at terminals, resulting in potential difference.

As shown in Figure 4b, the thermovoltage values of ITEC were measured under different temperature differences in the underwater environment. It can be seen that the thermovoltage rises up in multiples with increasing temperature difference, because the thermovoltage value is proportional to the temperature difference value. This result also illustrates that the TEIG-based ITEC can harvest heat energy to generate electrical energy successfully, even in the underwater environment. It should be noted that the thermoelectric properties of TEIG include sensitivity to humidity. As shown in Figure S11, the thermal voltage of TEIG increased slightly with the increase in relative humidity due to the adaptive aggregation of the hydrophobic network and ions in high humidity environments.

To deeply understand the operating process and mechanism of the TEIG-based ITEC, including harvesting low-grade heat energy underwater and powering external electrical appliances, the voltage variation of an external load of 5.6 kΩ connected with the underwater ITEC was recorded. As shown in Figure 4c, the four stages of voltage changing in one thermal cycle can be clearly identified, corresponding to the distribution stages of ions and electrons in the ITEC (Figure S12) [40,41]. Stage I is the thermoionic charging stage; driven by temperature gradient, cations and anions gather on the two electrodes and generate potential in the TEIG. However, since ions cannot reach the external circuit through the metal/ionogel interface, energy cannot be collected at this stage. Stage II is the forward electronic working stage; when the external load is connected with the ITEC, electrons flow through the external load to balance the charges on the two electrodes of the ITEC. The electrons driven by the thermovoltage of the TEIG layer act on the load in a manner similar to that of a supercapacitor [42,43]. Therefore, electricity collection can be realized at this stage. Because the charge on the two electrodes is balanced by the electrons and holes from the external circuit, the voltage between the two electrodes will decrease with time at this stage. At stage III, when the heat is cut off and the external load is disconnected, the thermoionic discharge stage comes. The cations and anions accumulated on the two electrodes dissipate. At the same time, due to the disconnection of the external circuit, the electrons of stage II still stay on the electrode. At the final stage (IV), the external load is reconnected to the ITEC to pull the electrons back, which causes the current to flow in the opposite direction to stage II. Therefore, this stage is called reverse electronic processing. In a typical thermal cycle, both stage II and IV can be used for electrical energy collection via switching on or off heat and connecting or disconnecting loads. The successful preparation of ITEC shows the advantages of simultaneous thermoelectric conversion and energy storage [44].

To cope with and adapt to more realistic and complex water environments, the thermovoltage of the TEIG-based ITEC was investigated in the simulated water that contains different concentrations of NaCl. As shown in Figure 4d, it is obvious that with the increase in NaCl content, the thermovoltage becomes continuously larger under the same temperature difference (10 K), varying from 23.3 mV of 0 wt % NaCl to 46.1 mV of 4 wt % NaCl. To explore the mechanism of this phenomenon, the density functional theory (DFT) was performed by calculating the interaction energy between different ions in the system of BMIMPF_6_ and NaCl. The chemical structures of different ions’ pairing include BMIM^+^: PF_6_^−^, BMIM^+^: Cl^−^, Na^+^: PF_6_^−^, and Na^+^: Cl^−^ (Figure 4e). The interaction energy between BMIM^+^ and Cl^−^ is larger than that between BMIM^+^ and PF_6_^−^, indicating that the combination of BMIM^+^ and Cl^−^ is more stable (Figure 4f). Therefore, when TEIG is immersed in an aqueous solution containing NaCl, BMIM^+^ that is exposed to water tends to combine with Cl^−^ to form [BMIM^+^]Cl^−^, so that more free PF_6_^−^ will appear, which further increases the difference in ion migration rate between BMIM^+^ and PF_6_^−^. Significantly, although NaCl can affect the dissociation and distribution of ionic liquid, it does not cause the leakage of ionic liquid, which was verified by detecting the change in the ionic conductivity of ionogel in the NaCl solution (Figure S13). The high thermovoltage in the NaCl solution attests that the hydrophobic TEIG-based ITEC can be used in most natural water areas, even in salt lakes and oceans.

Through previous work, we achieved underwater thermoelectric conversion by using TEIG to fabricate an underwater ionic thermoelectric capacitor; however, the thermovoltage generated by a single thermoelectric element is too small to meet the requirements as the power supply for external electrical equipment. To improve the output voltage of TEIG-based ITEC, a certain number of thermoelectric components can be assembled into one thermoelectric device to achieve multiple increases in thermovoltage. As shown in Figure 4g, the largest output voltage of 233.7 mV (∆*T* = 10 K) was observed by assembling five TEIG elements in series. The thermovoltage will continue to rise if the assembled number continues to increase, which provides the possibility for powering the external electrical devices that need high voltage.

Long-term stability is an important index to evaluate the reliability of devices in practical applications. A thermoelectric device was assembled by three TEIG elements in series, and its underwater cyclic thermoelectric performance under an intermittent temperature difference of 10 K was recorded. As shown in Figure 4h, the underwater thermovoltage maintained at about 81 mV in 100 time cycles, without obvious increase or decrease trends due to the superior water resistance of TEIG. Therefore, the thermoelectric device based on TEIG possesses excellent repeatability and thermovoltage stability in the underwater environment, proving the feasibility of long-term underwater operation.

### 3.4. Harvesting Low-Grade Heat under Water and in Rainy Weather

**Figure 5:**
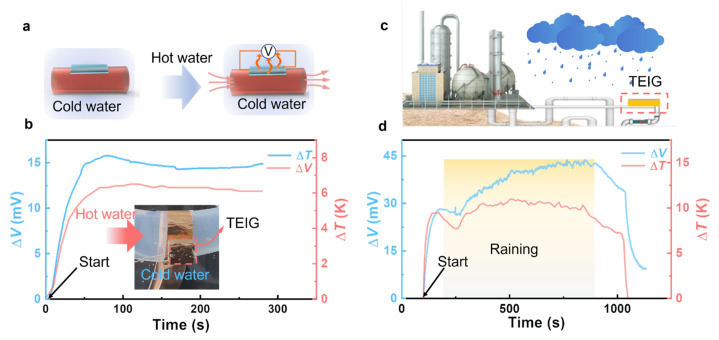
Demonstrations of ITEC harvesting waste energy in water environment: (**a**) Schematic of ITEC harvesting waste heat of industrial wastewater in underwater environment. (**b**) Variation of thermovoltage and temperature difference after hot water flowing into the pipe in water. (**c**) Schematic of ITEC operating in rainy environment. (**d**) Variation of thermovoltage and temperature difference in rain.

To study whether the thermoelectric device made of TEIG has the practicability of harvesting waste energy in a real water environment, some scenario demos were carried out in our research. The industrial wastewater with waste heat (generally above 50 °C) is usually discharged through the pipe into natural waters directly, resulting in the waste of heat. Considering the temperature difference between the hot pipe and the cold natural water with high specific heat capacity, it is expected to collect the waste heat of industrial wastewater by using the TEIG based on ITEC in the underwater environment. As shown in Figure 5a, the ITEC was attached to the outside surface of the hot pipe, which is immersed in cold water. Benefited by the good flexibility of the TEIG, the ITEC can be easily attached to the curved surface of the heat source. As shown in Figure 5b, when the pipe is full of hot wastewater, an increasing output voltage was observed along with the formation of the temperature difference within 90 s. Subsequently, the voltage remained relatively flat when the temperature difference was stable, indicating that the TEIG can be used to realize underwater thermoelectric power generation. The above results show that the hydrophobic TEIG has remarkable potential in harvesting low-grade heat energy under water in the future.

In addition to operation in an underwater environment, the TEIG-based ITEC can also operate normally on rainy days, owing to its excellent water resistance. As shown in Figure 5c, the ITEC was attached to the pipe surface in air, and an obvious output of thermovoltage could be observed, owing to a temperature difference being formed when hot water is passed in the pipe (Figure 5d). More importantly, the thermovoltage of the TEIG-based ITEC did not decay after raining. The good stability in rain provides evidence for the feasibility of all-weather operation.

## 4. Conclusions

In summary, a hydrophobic TEIG with excellent long-term underwater stability and reasonable thermoelectric performances was developed by one-step photopolymerization of hydrophobic monomer in hydrophobic IL solvent. The fully hydrophobic structure not only endows the TEIG with outstanding antiswelling ability through preventing the cross-interface diffusion of water molecules but also effectively inhibits the leakage of ions in the aquatic environment. Excellent water resistance enables the TEIG to maintain long-term stability under water, providing the foundation for underwater thermoelectric application. Furthermore, the ionic thermoelectric device fabricated by TEIG exhibited superior thermoelectric performance in the underwater environment, including thermopower, durability for underwater operation, and salt tolerance. More significantly, the water-resistant ionic thermoelectric device demonstrated the good stability and reliability expected in both underwater and rainy environments, showing potential for all-environment and all-weather operation. The TEIG broadens the application scope of TE materials and provides an effective approach to harvest underwater low-grade heat energy in the future, which is significant for alleviating the energy crisis and promoting sustainable development.

## Data Availability

The data that support the findings of this study are available from the corresponding author.

## Supplementary Materials

The following supporting information can be downloaded at: https://www.mdpi.com/article/10.3390/polym15071746/s1, Figure S1: Chemicals structures used in the experiment; Figure S2: Schematic illustration for the ionic thermoelectricity measurement device; Figure S3: Mixed solution of BMIMPF_6_ and water (add colorant); Figure S4: FTIR spectra of the resulting ionogel after polymerization; Figure S5: Water contact angle (WCA) of the TEIGs with different MMA content; Figure S6: Tensile curves of the TEIGs with different MMA content; Figure S7: Comparison of tensile properties of TEIG (the volume ratio of IL and MMA is 4:1) before and after soaking in water for 1 day; Figure S8: Thermogravimetric curves of the TEIGs with different MMA volume content; Figure S9: linear fitting of the Δ*V*-Δ*T* plots of TEIG with a volume ratio of IL and monomer of 4:1; Figure S10: Comparison in ionic Seebeck coefficient of quasi-solid ionogel materials; Figure S11: Thermal voltage of TEIG under different relative humidity (∆*T* = 10 K); Figure S12: (a) Illustration of the working principle of ITEC in the four stages. There are four stages in one cycle. Stage I: Thermoionic charging; stage II: forward electronic working; stage III: thermoionic discharging; stage IV: reverse electronic working. (b) Thermoelectricity (TE) conversion of an ionic thermoelectricity capacitors (ITESC) in a full thermoelectrici cycle under temperature gradient (∆*T*). (c) Schematic diagram of TEIG-connected external load and voltage test circuit; Figure S13: Ionic conductivity change of the hydrophobic ionogel after soaking in NaCl solution (3 wt %).
