# Supplementary material for: Water-Resistant Thermoelectric Ionogel Enables Underwater Heat Harvesting
Source: Polymers (Basel). 2023 Mar 31;15(7):1746. doi: 10.3390/polym15071746 (PMC10097396; doi:10.3390/polym15071746)
Supplement: Supplementary file 1 [file polymers-15-01746-s001.zip › polymers-2263029-supplementary.pdf]

polymers

MDPI

Supporting Information

Water-resistant thermoelectric ionogel enables underwater heat-harvesting

Long Li[†1,2], Huijing Li[†2,3], Junjie Wei [2,3,*], Rui Li[1,2], Jiale Sun[2,3], Chuanzhuang Zhao[1,*],Tao Chen[2,3,*]

[1] School of Material Science and Chemical Engineering, Ningbo University, Ningbo, 315211, China.
[2] Key Laboratory of Marine Materials and Related Technologies, Zhejiang Key Laboratory of Marine Materials and Protective Technologies, Ningbo Institute of Material Technology and Engineering, Chinese Academy of Sciences, Ningbo 315201, China
[3] School of Chemical Science, University of Chinese Academy of Sciences, 19A Yuquan Road, Beijing 100049, China.
* Correspondence: weijunjie@nimte.ac.cn (J.W); zhaochuanzhuang@nbu.edu.cn (C,Z); tao.chen@nimte.ac.cn (T,C)
[†] These authors contribute equally to this work.

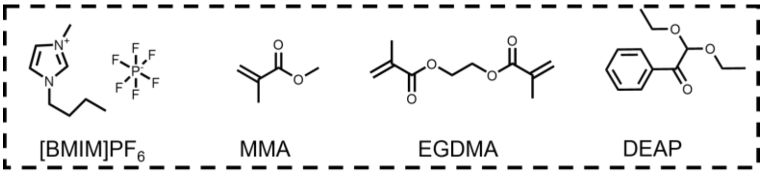

Figure S1. Chemicals structures used in the experiment.

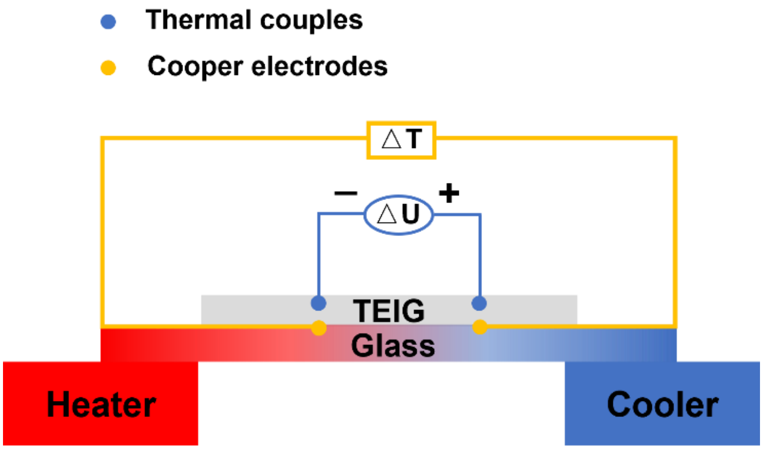

Figure S2. Schematic illustration for the ionic thermoelectricity measurement device.

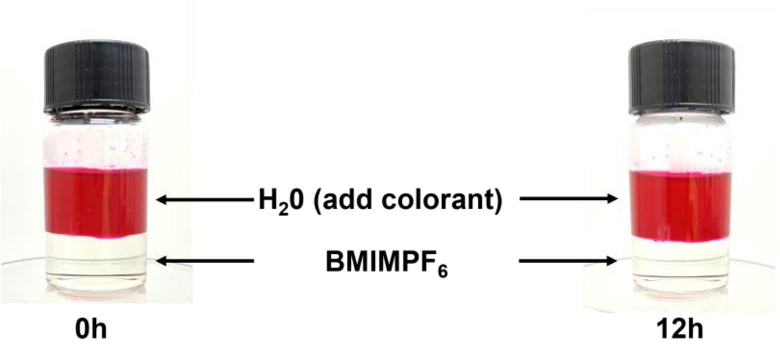

Figure S3. Mixed solution of $BMIMPF_6$ and water (add colorant).

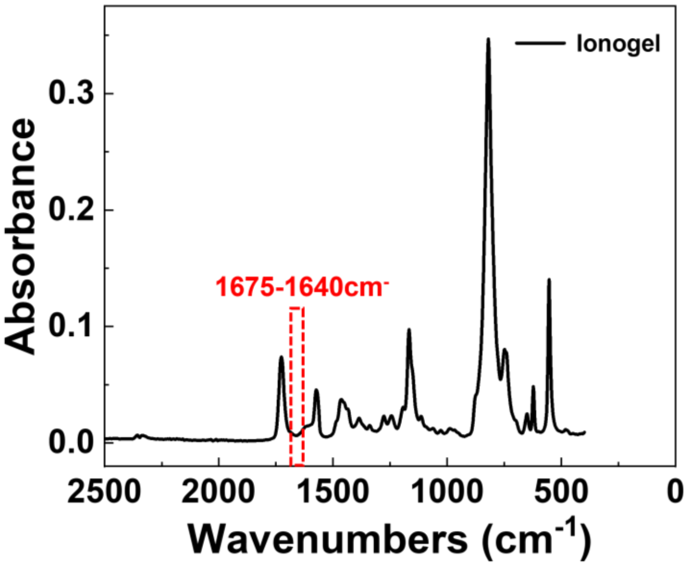

Figure S4. FTIR spectra of the resulting ionogel after polymerization.

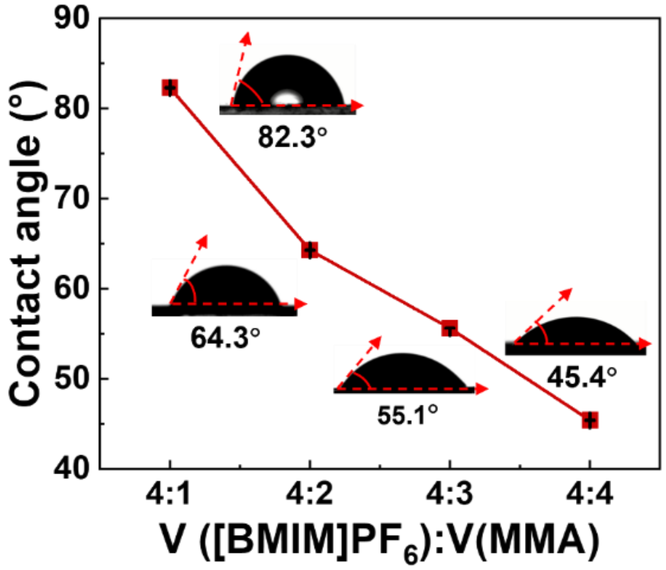

Figure S5. Water contact angle (WCA) of the TEIGs with different MMA content.

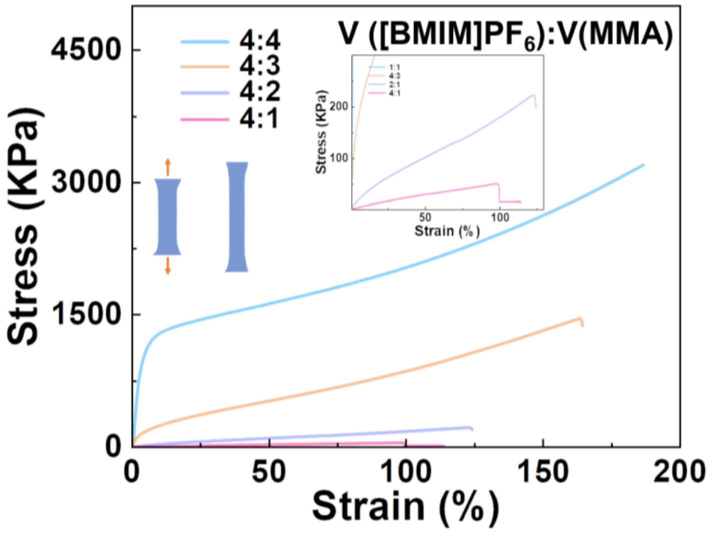

Figure S6. Tensile curves of the TEIGs with different MMA content.

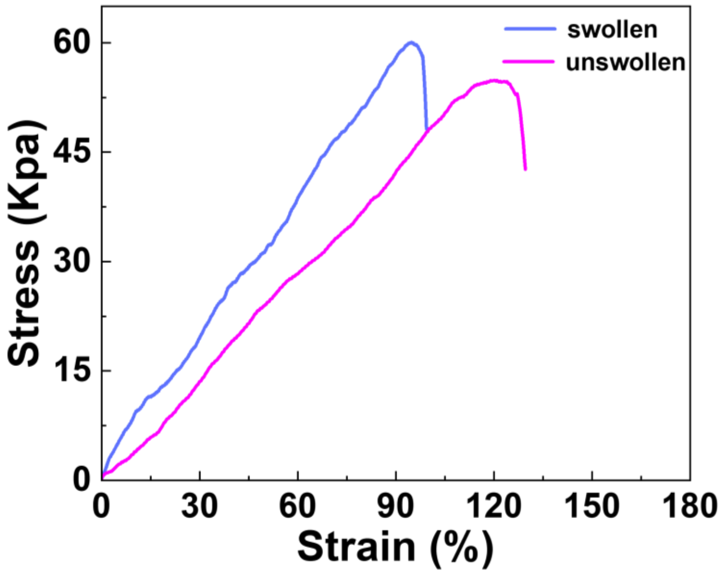

Figure S7. Comparison of tensile properties of TEIG (the volume ratio of IL and MMA is 4:1) before and after soaking in water for 1 day.

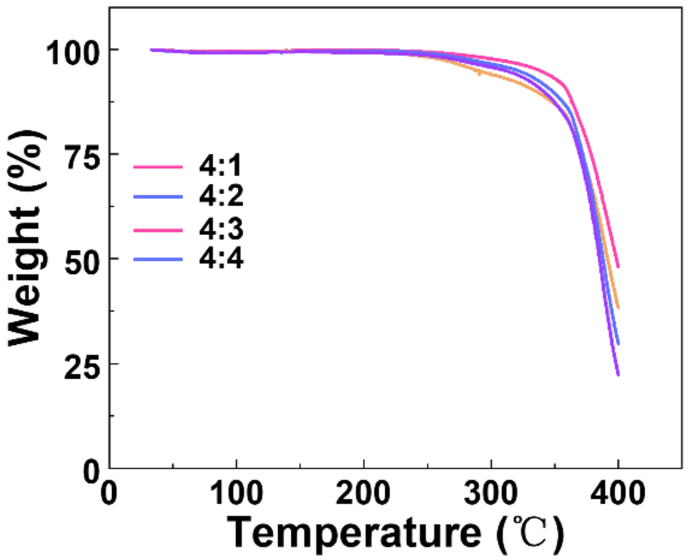

Figure S8. Thermogravimetric curves of the TEIGs with different MMA volume content.

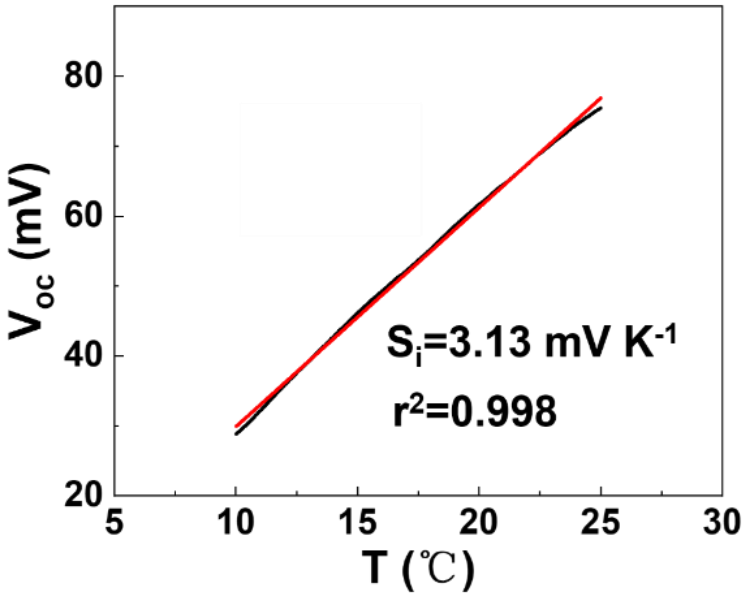

Figure S9. linear fitting of the $\Delta V - \Delta T$ plots of TEIG with the volume ratio of IL and monomer being 4:1

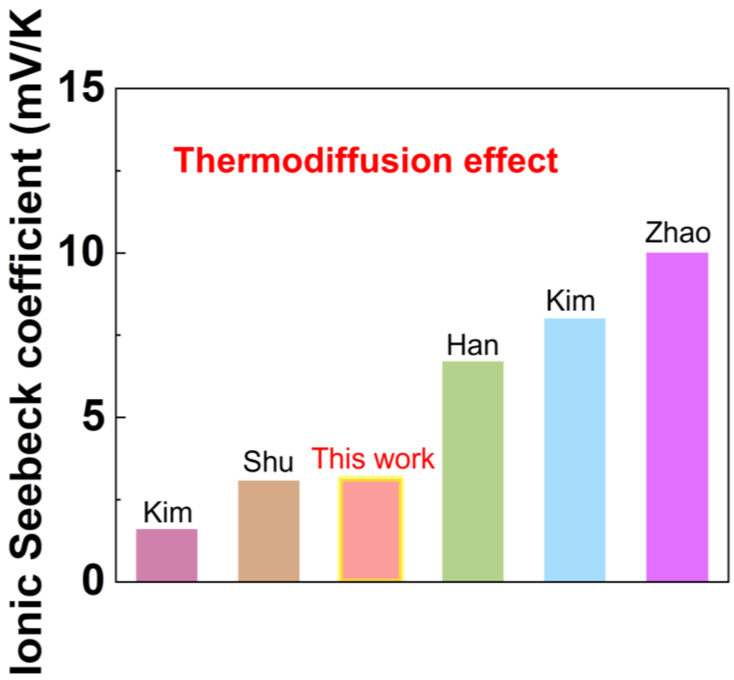

Figure S10. Comparison in Ionic Seebeck coefficient of quasi-solid ionogel materials.

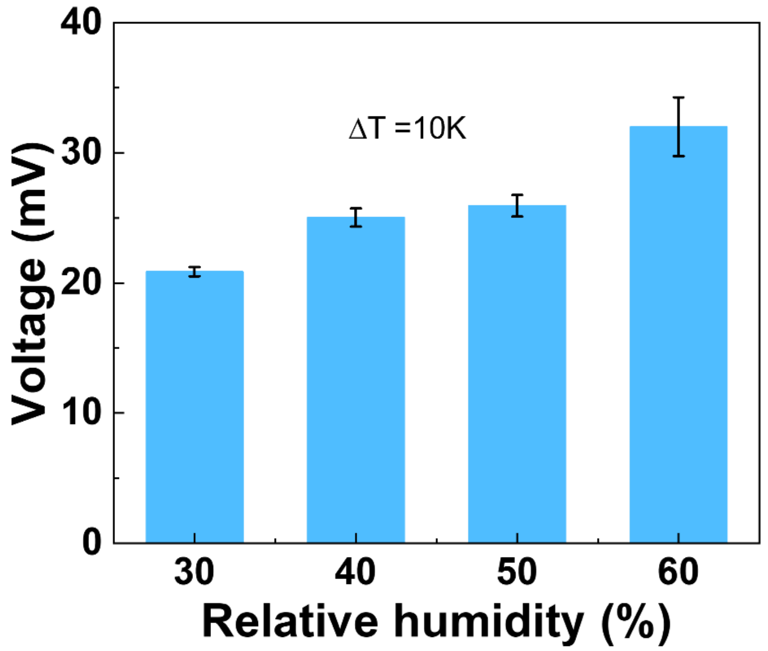

Figure S11. Thermal voltage of TEIG under different relative humidity (ΔT = 10 K).

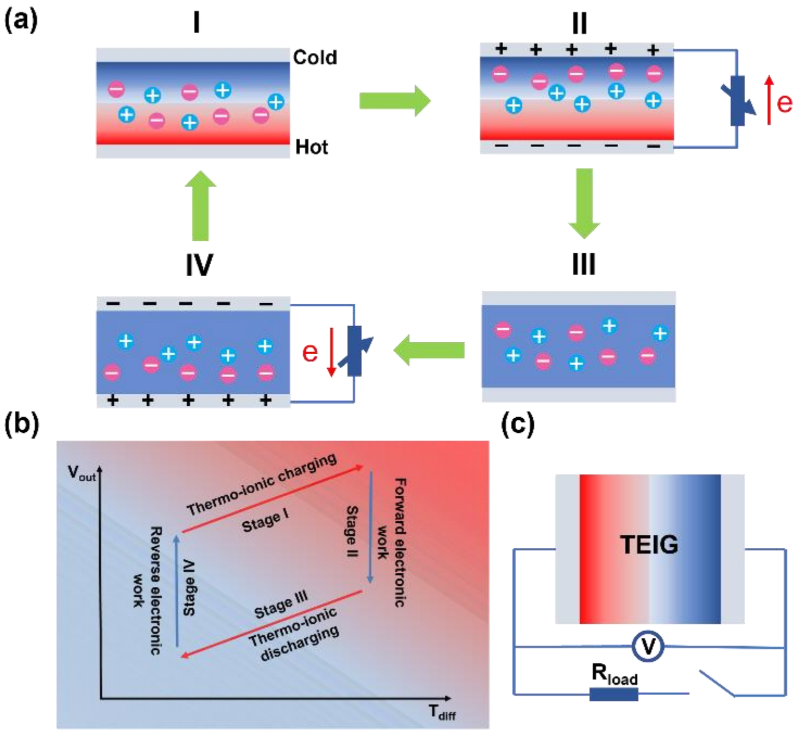

Figure S12. (**a**) Illustration of the working principle of ITEC in the four stages. There are four stages in one cycle. Stage I: Thermo-ionic charging; stage II: forward electronic working; stage III: thermo-ionic discharging; stage IV: reverse electronic working. (**b**) Thermoelectricity (TE) conversion of an ionic thermoelectricity capacitors (ITESC) in a full thermal-electricity cycle under temperature gradient(ΔT). (**c**) Schematic diagram of TEIG connected external load and voltage test circuit.

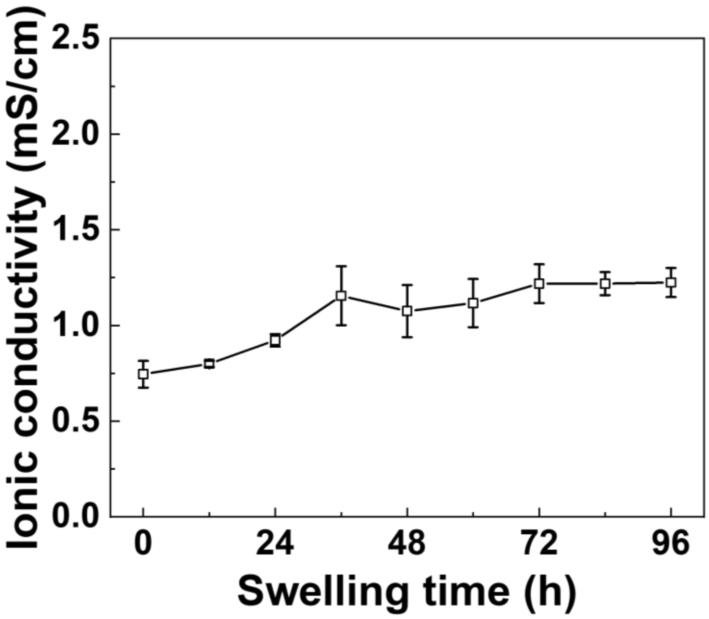

Figure S13. Ionic conductivity change of the hydrophobic ionogel after soaking in NaCl solution (3 wt%).
